# Prevalence of unwanted pregnancy in Iranian women: a systematic review and meta-analysis

**DOI:** 10.1186/s12978-019-0804-8

**Published:** 2019-09-04

**Authors:** Rostam Jalali, Masoud Mohammadi, Aliakbar Vaisi-Raygani, Akram Ghobadi, Nader Salari

**Affiliations:** Department of Nursing, School of Nursing and Midwifery, Kermanshah University of Medical Sciences, Kermanshah, Iran

**Keywords:** Prevalence, Unwanted pregnancy, Women, Iran, Meta-analysis

## Abstract

**Background:**

Unwanted pregnancies are considered as one of the most important public health risks. Regarding the importance of the unwanted pregnancy in the country and helping health policy-makers obtain more accurate information on this issue, this study aims to provide a systematic review and meta-analytical on the prevalence of unwanted pregnancies in Iran.

**Methods:**

The present study was carried out using meta-analysis. Articles related to the topic were obtained through SID, Magiran, Scopus, PubMed, and ScienceDirect and Google Scholar databases from 2001 to 2017, Articles written based on cross-sectional studies were included in the study and other overviews, case-control, cohort, and interventional studies were excluded from the list of articles. Heterogeneity of studies was investigated using I^2^ index and data analysis was performed in Comprehensive Meta-Analysis software (Version 3).

**Results:**

In 23 articles, the prevalence of unwanted pregnancy in Iranian women was 27.9% (95%CI: 24–32.1%). The meta-regression analysis was used in two sample sizes and years of study. It was reported that as the sample size and Years increases, the prevalence of the unwanted pregnancy decrease, this difference was also statistically significant (*P* = 0.000).

**Conclusion:**

Considering that the prevalence of unwanted pregnancy is high in Iran, it is necessary that health policy makers take effective measures to enhance the awareness of couples and public information about the risks of the unwanted pregnancy.

## Plain English summary

Unwanted pregnancies are considered as one of the most important public health risks. Given that the overall outbreak in Iran is unclear, the present study raised the question what is the overall Prevalence of Unwanted Pregnancy in Iranian Women throughout the country? The present study was carried out using meta-analysis. Articles related to the topic were obtained through SID, Magiran, Scopus, PubMed, and ScienceDirect and Google Scholar databases from 2001 to 2017, Articles written based on cross-sectional studies were included in the study and other overviews, case-control, cohort, and interventional studies were excluded from the list of articles. In 23 articles, the prevalence of unwanted pregnancy in Iranian women was 27.9%, Considering that the prevalence of unwanted pregnancy is high in Iran, it is necessary that health policy makers take effective measures to enhance the awareness of couples and public information about the risks of the unwanted pregnancy.

## Background

The increasing population growth is one of the most important barriers to economic growth, social progress, and a decrease in the promotion of the health level in modern countries. On the other hand, 50% of births have been unplanned and 25% of births have been unwanted pregnancies [1], family planning plays a very important role in controlling population and social balance in society, while unwanted and unplanned pregnancies are one of the most important public health risks [2].

Unwanted pregnancy is a pregnancy that is usually accidental and is not requested by one or both parents. According to the conducted studies, 120 million women in developing countries have unwanted pregnancy because of not using family planning methods, this means that one out of five pregnancies is unwanted [2, 3]. Reports show that in 2008, there were 22 million unsafe abortions due to unwanted pregnancy, resulted in more than 47,000 mothers’ mortalities (13%) [4]. In Iran, despite the availability of contraceptive methods, a significant proportion of pregnancies (more than 25 to 35%) is still unplanned in different areas due to the use of traditional methods or irregular and incorrect use of preventive methods, and 4000–5000 unwanted pregnancies are occurred annually, out of which 16% of cases lead to abortion. In spite of government’s efforts for family planning, only 55.4% of family planning equipment have been used, and various studies have reported the incidence of unwanted pregnancy in the range of 18% to over 35% [4–6]. Such a situation leads to the reduction of the quality of life, mother’s mental disorders, suicide, the occurrence of risky behaviors such as smoking and using alcohol in the mothers [7].

Unwanted pregnancy and its complications is a global issue that affects women, families and the community, and in most cases leads to deliberate abortion or even mother’s death. Therefore, the reduction of unwanted pregnancy is followed by reducing maternal mortality in one hand and, reducing hospital burden and costs on the other hand. It can also have a positive effect on improving the level of welfare of couples and the pattern of population growth and economic and social development of society are guaranteed [8, 9].

Given that the overall outbreak in Iran is unclear, present study raised the question what is the overall Prevalence of Unwanted Pregnancy in Iranian Women throughout the country?

Therefore, regarding the importance of the unwanted pregnancies in Iran and helping health policymakers obtain more accurate information on this topic, this study aims to provide a systematic and meta-analysis review on the prevalence of the unwanted pregnancies in Iran.

## Methods

This study was a systematic review and meta-analysis and it is the result of the findings extracted from studies on the prevalence of unwanted pregnancy in Iranian women; and the articles included were published either in domestic or foreign journals and the search in Magiran, SID, Medline (PubMed) and ScienceDirect, Scopus and Google scholar databases was limited to articles conducted within March, 2001 to October, 2017.

### Search strategy

The database search process was performed through the use of keywords in Persian, and also through English equivalent of the keywords, i.e., pregnancy, unwanted pregnancy, Iran and their possible combinations. It means that the keywords in Persian were used in search process in Farsi databases and the English equivalents of the keywords were used on English databases; moreover, both keywords in Persian and the English equivalents as mentioned above were used to search in Google scholar search engine and the operators AND, OR were used to search keywords in combination so that to gain full access to all articles. Hence, the operator OR was used to check the common names used for one disorder, e.g., (((((Pregnancy [Title/Abstract]) OR Fertilization [Title/Abstract]) AND unwanted pregnancy [Title/Abstract]) OR Pregnancy Unplanned [Title/Abstract]) AND Women [Title/Abstract]) OR Female [Title/Abstract]))))) through matching the search term with items on MeSH browser.

### Articles selection and evaluation criteria

First, all articles were collected using the selected keywords and a list of abstracts was prepared after the search was completed. After concealing the profile of the articles, including the name of the journal and name of the author, the full texts of the articles were submitted to the reviewers. Each article was read independently by two reviewers. If the article was rejected, the reason for rejection was mentioned and in cases of disagreement between the two reviewers, the article is evaluated by a third reviewer and the opinion of the third reviewer is taken into account. Articles were in Persian and English and were obtained from cross-sectional studies on the prevalence of unwanted pregnancy in Iranian women and they met the selection criteria to be included in the study, and other review, case-control, cohort, and interventional studies were excluded from the list of articles. In this study, in order to review Gray Literature, i.e., part of the evidence and documentation that was not published or distributed for any reason, a general search on the Google search engine and the review of the related sites were also on the agenda.

### Statistical analysis

In each study, the prevalence of unwanted pregnancy in Iranian women was obtained. *I*^*2*^ test was used to examine the heterogeneity of studies. Generally, heterogeneity is classified as three categories: heterogeneity less than 25% (low level of heterogeneity), between 25 and 75% (average level of heterogeneity) and more than 75% (high level of heterogeneity). Data were analyzed using Comprehensive Meta-Analysis software (Biostat, Englewood, NJ, USA version 3). The probability of publication bias in results was measured using the funnel plot, the Egger test and the significance level of 0.05. Also, the meta-regression test in two factors of the sample size and research year was used to investigate the effects of the potential factors affecting the heterogeneity of the studies.

## Results

### Search output

**Fig. 1:**
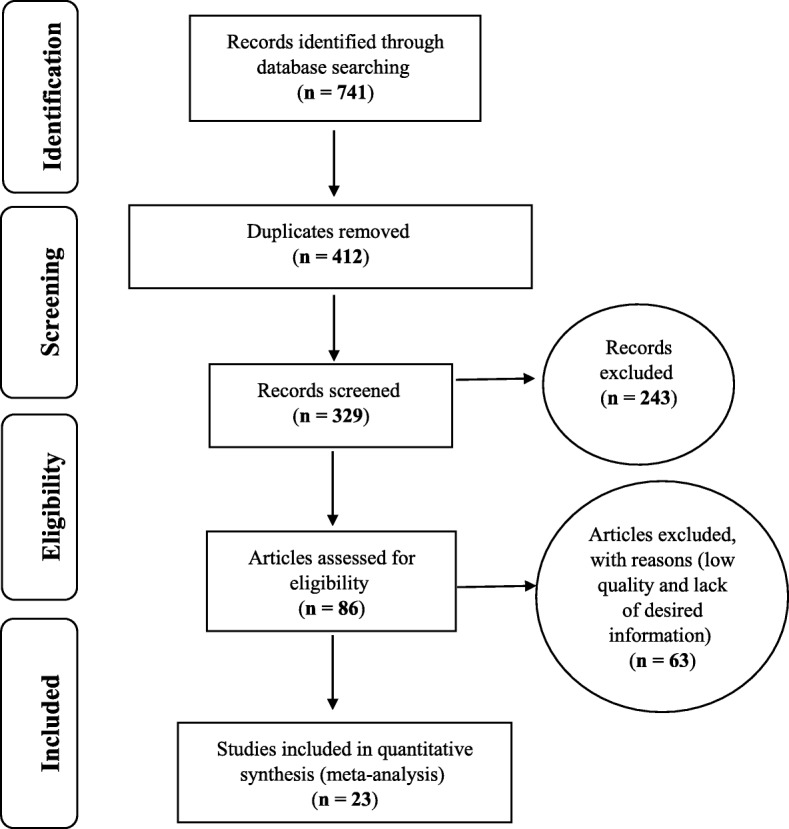
the flowchart on the stages of including the studies in the systematic review and meta-analysis

The study review process was reported based on the PRISMA 2009 process (Fig. 1). A table was prepared that contains information from selected articles including the name of the researcher, the title of the article, the year and place of the study, the number of samples, and the prevalence of unwanted pregnancy in studies (Table 1). Eventually, in the final review, the related articles were entered the meta-analysis stage that as a result 23 appropriate articles were entered to this stage [10–32].

**Table 1:** Specifications of studies entered the stud

Row	Author [References]	Published	Area	Age	Sample size	Prevalence
1	Kiani [10]	2013	Mashhad	34.9 ± 5.6	105	29.5
2	Hassan-Ghasemi [11]	2013	Gorgan	32.1 ± 7.4	339	13
3	Abdullahi [12]	2004	Mazandaran	18–35	1200	10.6
4	Amin shokravi [13]	2004	Tehran	10–49	150	31.3
5	Zaheri [14]	2014	Sanandaj	20–40	1070	25.1
6	Ostadnowrozi [15]	2012	Kurdistan	18–36	1134	25.1
7	Mohammadpourasl [16]	2005	Tabriz	26 ± 5.4	1576	26.7
8	Sereshti [17]	2005	Shahr e Kord	25.2 ± 5.3	1925	27.2
9	Azizi [18]	2011	Kermanshah (Salas e Babajani)	25.7 ± 4.1	102	40.2
10	Jahanfar [19]	2002	Iran (Fars, Sistan and Baluchestan, Hamedan, Kerman, Mazandaran, Gilan, Khorasan, Khuzestan, Isfahan and West Azarbaijan)	29.6 ± 7.1	1548	34.9
11	Shahbazi [20]	2006	Semnan	20–35	405	25.7
12	Kahnamoeiaghdam [21]	2005	Ardebil	13–44	600	30.8
13	Zamani [22]	2005	Isfahan (Najafabad)	24.9 ± 5.1	788	42
14	Jarahi [23]	2014	Khorasan Razavi (Sarakhs)	18–35	300	21.7
15	Namazi [24]	2014	Rasht	27.5 ± 4.6	270	49.3
16	Shahbazin [25]	2015	Kermanshah (Kangavar)	31.3 ± 7.4	248	21.2
17	Amani [26]	2010	Ardebil	28.2 ± 6.7	328	60.7
18	Mirzamoradi [27]	2017	Tehran	15–45	950	19.2
19	Tavakoli [28]	2002	Rafsanjan	–	356	38.8
20	Kazemi [29]	2001	Zanjan	20–30	500	28
21	Mohammadsalehi [30]	2010	Arak	–	352	27.8
22	Vakili [31]	2010	Yazd	28.3 ± 5.8	330	22.9
23	Vizshafar [32]	2005	Fars (Grash and Lar)	26.4 ± 5.4	500	18.1

Based on studies on the prevalence of unwanted pregnancy in Iranian women that included articles published either in domestic or foreign journals, and through searching Magiran, SID databases 39 articles, Medline (PubMed) 138 articles and Science Direct 362 articles, Scopus 62 articles and Google Scholar 140 articles were obtained; then articles that met the initial inclusion criteria were included in the study but via primary assessment, the exclusion of 412 duplicate ones left 329 articles that by elimination of another 243 articles unrelated to subject of study and the elimination of 63 articles in a secondary assessment due to the lack of access to the abstract and full text of the articles and because of the poor quality of the articles eventually 23 articles entered the meta-analysis process, the total number of participants in the study was 15,076 individuals aged between 10 and 50 years old, the highest sample size was in the study of Serehti et al. [17] in Shahrekord (1925 individuals) and the lowest sample size (102) in the study of Azizi et al. [18] in Kermanshah (Salas Babajani).

### Heterogeneity and publication bias

**Fig. 2:**
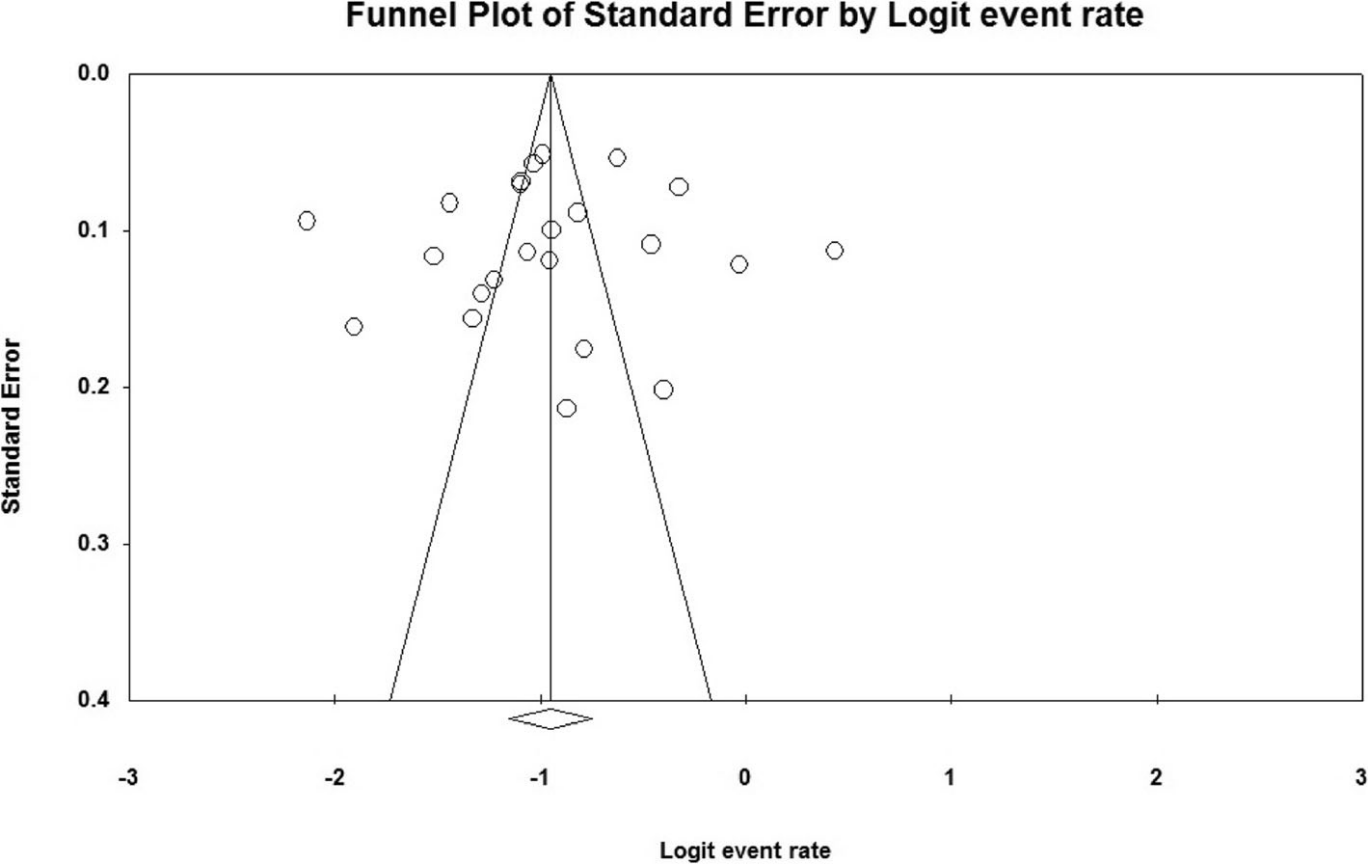
Funnel Plot. Results of unwanted pregnancy prevalence in Iranian women

The heterogeneity of the studies was evaluated using the I^2^ test which value was 96.5% showing a high heterogeneity in the studies. Therefore, the random effects model was used to combine the results of the studies. The publication bias was evaluated via funnel plot and Egger’s test at a significance level of less than 0.05, which the publication bias was not statistically significant (*P* = 0.402) (Fig. 2).

### Meta-analysis

**Fig. 3:**
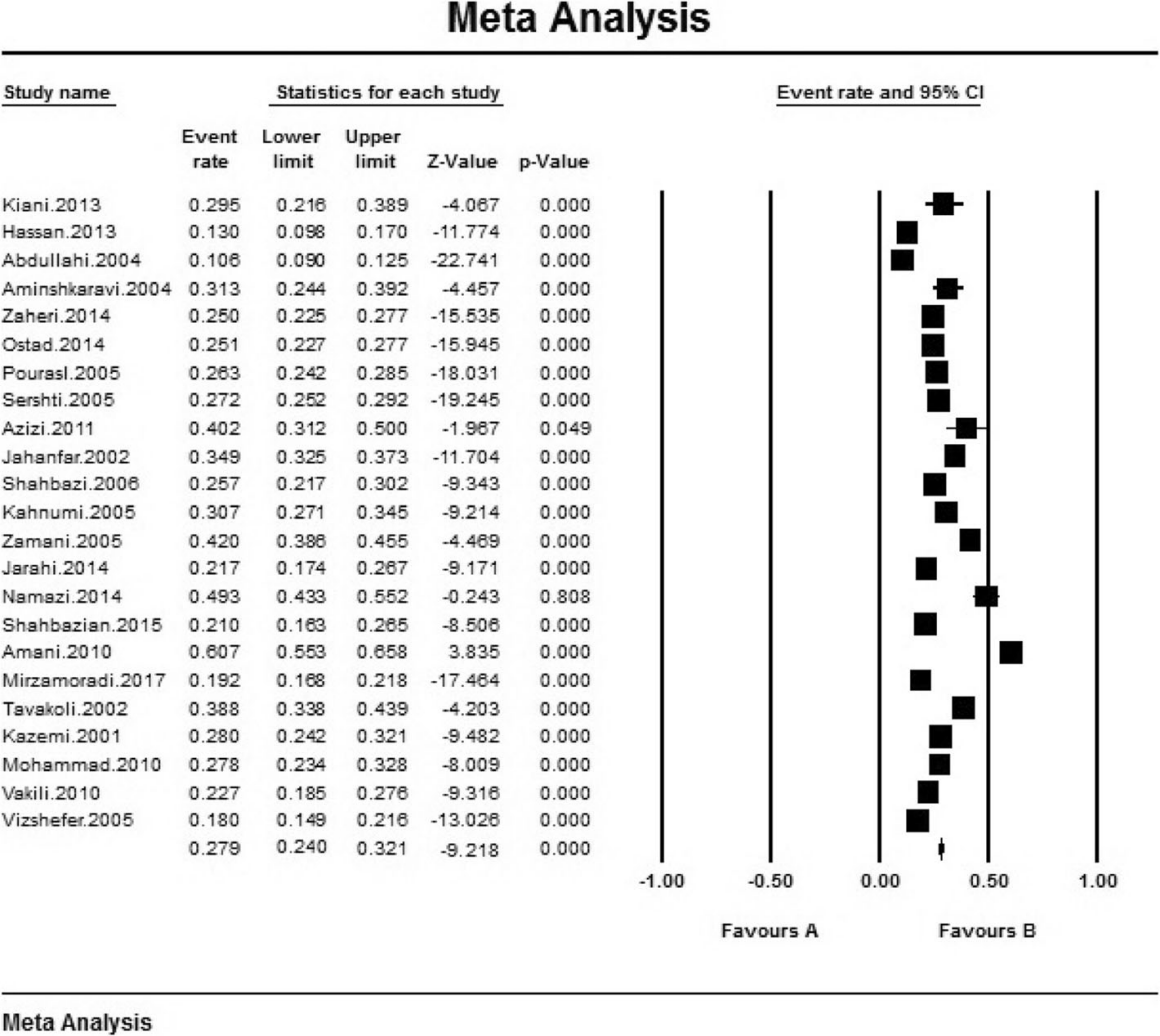
The Prevalence of Unwanted Pregnancy in Iranian Women Based on Randomized Model

Based on the random effects model, it has been shown that the black square is the prevalence rate and the length of the line segment on which the square places is 95% confidence interval in each study, the rhubarb shows the prevalence rate throughout the country for the whole study. Accordingly, the overall prevalence of unwanted pregnancy in Iranian women is 27.9% (95% CI: 24.1–32%), the highest prevalence of unwanted pregnancy was in Ardebil with 60.7% (95% CI: 3.8–55.65%) [26] And the lowest prevalence of unwanted pregnancy was in Mazandaran with 10.6% (95% CI: 9.5–12%) [12] (Fig. 3).

### Sub-group analysis

Table 2 reports the results of the different sub-group analysis according to the Years of publication, and Sample size (Table 2).

**Table 2:** The results of sub-group analyses

Variables		No. studies	Prevalence% (95% CI)	I^2^ (%)	*P* value	No. participants
Years	2001–2006	11	27.5 (22.5–33.1)	96.8	0.000	9548
	2007–2012	5	34.3 (21.9–49.3)	97.3	0.000	2246
	2013–2017	7	24.3 (17.8–32.3)	95.1	0.000	3282
Sample size	≤500	14	29.3 (23–36.7)	95.6	0.000	4285
	500–1000	3	29.8 (18–45)	98	0.000	2338
	1000<	6	24 (18.7–30.3)	97.4	0.000	8453

### Meta-regression

**Fig. 4:**
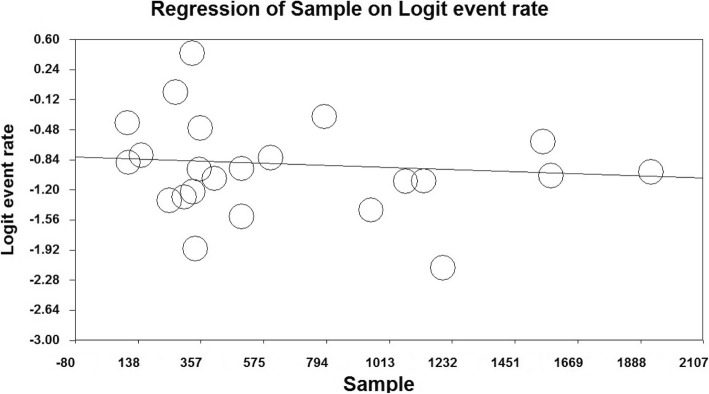
Meta-regression of Unwanted Pregnancy Frequency in Iranian Women based on Sample Size

**Fig. 5:**
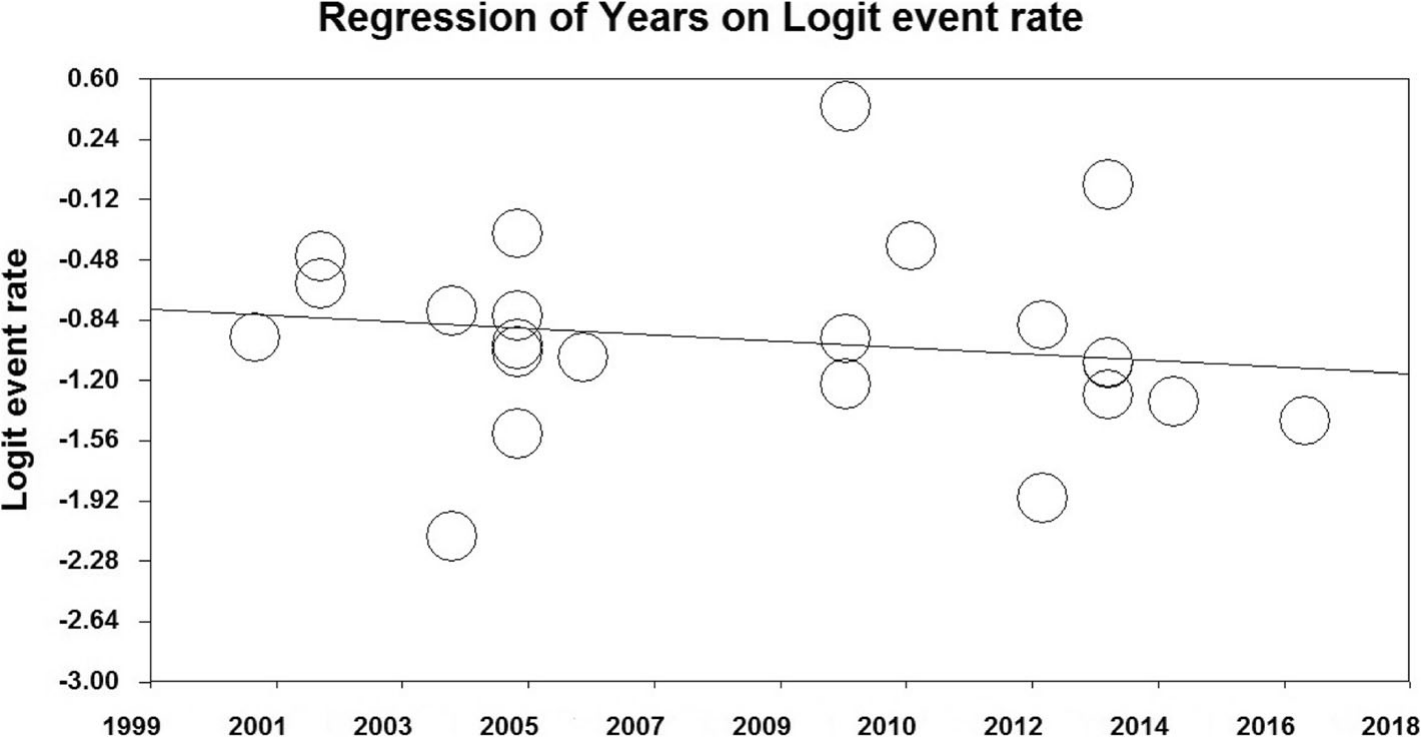
Meta-regression of Unwanted Pregnancy Frequency in Iranian Women based on the Year of Study

In order to investigate the effects of potentially effective factors on heterogeneity in the prevalence of unwanted pregnancy in Iranian women, meta-regression was used for two factors of sample size and the year of study (Figs. 4 and 5). The prevalence of the unwanted pregnancy also decreases with increasing sample size in the studies, and this difference was statistically significant (*P* = 0.000) (Fig. 4). It was also reported in (Fig. 5) that, as the year of the study progresses, the frequency and prevalence of unwanted pregnancy decrease. This difference was also statistically significant (*P* = 0.000).

## Discussion

Unwanted pregnancy, in addition to imposing economic costs on the whole community, affects the health of the mother and her survival. During pregnancy, the mother may suffer from pregnancy complications, especially anemia, and may be at risk of various diseases that affect her physical and mental health [30–32]. According to the World Health Organization (WHO), over one-third of the pregnancies were reported unwantedly and most unwanted pregnancies are related to the developing countries [33], 60% were reported in the US study [34], while in another study in the United States, 17% of unwanted pregnancies have been over 35 years old [35]. In addition, in the U.S national survey [36] and in the Lawrence [37] and Ventura’s [38] studies in 2011 and 2012 in the United States, the prevalence of unwanted pregnancy has been reported to be 49%, in Kenya this prevalence is 24% [39], in Ethiopia 36.5% [40], The unwanted pregnancy prevalence in Nepal [41], the unwanted pregnancy rate of 68% of cases per 1000 women was in the fertility age.

Of factors affecting unwanted pregnancy are the age of the husband with an age difference of 4 or more years, the marriage age, the age of the first intercourse, the lack of knowledge and the use of contraceptive methods, the low economic and social situation. In the results of this study, the unwanted pregnancies were at older ages and studies have shown that husband’s age has a significant relationship with the prevalence of unwanted pregnancy and this relationship is inverse indicating the fact that increasing husband’s age and unwanted pregnancy by the husband and on the other hand his failure to cooperate with his wife to carry out safe prevention services lead to an increase in unplanned pregnancies [26, 27]. The unwanted pregnancy, in addition to maternal health and the risk of abortion, afflicts the mother’s mental health and leads to mental illness and even suicide [26]. Various studies have reported that 60% of suicides in pregnant women have been in the third trimester and after knowing about the unwanted pregnancy [26–29]. The unwanted pregnancy is one of the important indicators of quality assessment of family planning services, which can endanger the pregnancy health as it was mentioned. The unwanted pregnancy across the world is a threat for mother and child health due to the lack of family support and it is among the causes that increase mothers’ mortality and a major obstacle to the improvement of reproductive and sexual health [13]. Compared to other children, children born in unwanted pregnancies are premature with low birth weight and they use breastfeeding less [42]. Unwanted children are more likely to be bothered or neglected by parents and will have a more limited emotional connection with the mother. Also, such conditions make these children have low self-confidence [23]. Unwanted pregnancy is also one of the causes of a large increase in the size of the population, which makes housing, educational space, food shortages, annual income decline, inflation, poor quality of health care, the increasing overhead, the increasing unemployment rate. Therefore, in order to achieve the goals of social, economic and cultural development, it is necessary to improve modernization programs, social justice, a proper coordination between population and the development programs [43].

According to reports, 75 million out of 175 million registered pregnancies were unwanted worldwide, and according to the World Health Organization reports, such conditions make it possible to witness about 20 million dangerous abortions, resulting in deaths of 60–100 thousand mothers per year. In Iran, about 80,000 intentional abortions have been reported annually due to the unwanted pregnancy, many of which result in maternal death or inability due to unsafe abortions. Considering that the prevalence of unwanted pregnancy is high and the unwanted pregnancies are mainly due to family planning inappropriate behaviors, one third-one fourth of mothers’ mortalities can be prevented by the correction of family planning behaviors and the increase of awareness of couples regarding the risks of unwanted pregnancies [43–45]. Many unwanted pregnancies are reduced and prevented by enhancing men’s awareness, especially at older ages, and with the improvement of these conditions, it is possible to reduce the number of abortions due to the unwanted pregnancies and increase the welfare of the community.

### Strengths and limitations

One of the strengths of the present study was the provision of an overall Prevalence of Unwanted Pregnancy in Iranian Women for the first time. Moreover, meta-regression was used in this study for the two factors of sample size and publication year. On the other hand, the most important limitations of the study were related to the inaccessibility of the full-text of some retrieved studies and the lack of information required in some studies.

## Conclusion

Considering that in Iran the prevalence of unwanted pregnancy is high, health policy makers need to take effective measures to increase awareness among couples and publicly inform about the risks of the unwanted pregnancy.

## Data Availability

Datasets are available through the corresponding author upon reasonable request.

## Abbreviations

PRISMA: Preferred Reporting Items for Systematic Reviews and Meta-Analysis
SID: Scientific Information Database
STROBE: Strengthening the Reporting of Observational Studies in Epidemiology for cross- sectional Study
WHO: World Health Organization
